# Phosphorylation-mediated disassembly of C-terminal binding protein 2 tetramer impedes epigenetic silencing of pluripotency in mouse embryonic stem cells

**DOI:** 10.1093/nar/gkae1076

**Published:** 2024-11-26

**Authors:** Han-Teo Lee, Young Ah Kim, Sangho Lee, Ye-Eun Jung, Hanbyeol Kim, Tae Wan Kim, Sojung Kwak, Jaehyeon Kim, Chul-Hwan Lee, Sun-Shin Cha, Jinmi Choi, Eun-Jung Cho, Hong-Duk Youn

**Affiliations:** Stochastic Stemness Research Center, Department of Biomedical Science, Seoul National University College of Medicine, Seoul 03080, Republic of Korea; Ischemic/Hypoxic Disease Institute, Seoul National University Medical Research Center, Seoul 03080, Republic of Korea; Stochastic Stemness Research Center, Department of Biomedical Science, Seoul National University College of Medicine, Seoul 03080, Republic of Korea; Stochastic Stemness Research Center, Department of Biomedical Science, Seoul National University College of Medicine, Seoul 03080, Republic of Korea; Department of Chemistry and Nanoscience, Ewha Womans University, Seoul 03760, Republic of Korea; Stochastic Stemness Research Center, Department of Biomedical Science, Seoul National University College of Medicine, Seoul 03080, Republic of Korea; Department of Pharmacology, Cancer Research Institute, Seoul National University College of Medicine, Seoul 03080, Republic of Korea; Department of Interdisciplinary Engineering, Daegu Gyeongbuk Institute of Science and Technology (DGIST), Daegu 42988, Republic of Korea; Developmental Biology Laboratory, Environmental Disease Research Center, Korea Research Institute of Bioscience and Biotechnology, Daejeon 34141, Republic of Korea; Stochastic Stemness Research Center, Department of Biomedical Science, Seoul National University College of Medicine, Seoul 03080, Republic of Korea; Stochastic Stemness Research Center, Department of Biomedical Science, Seoul National University College of Medicine, Seoul 03080, Republic of Korea; Ischemic/Hypoxic Disease Institute, Seoul National University Medical Research Center, Seoul 03080, Republic of Korea; Department of Pharmacology, Cancer Research Institute, Seoul National University College of Medicine, Seoul 03080, Republic of Korea; Department of Chemistry and Nanoscience, Ewha Womans University, Seoul 03760, Republic of Korea; R&D Division, TODD PHARM CO. LTD., Seoul 03760, Republic of Korea; School of Pharmacy, Sungkyunkwan University, Suwon 16419, Republic of Korea; School of Pharmacy, Sungkyunkwan University, Suwon 16419, Republic of Korea; Stochastic Stemness Research Center, Department of Biomedical Science, Seoul National University College of Medicine, Seoul 03080, Republic of Korea; Ischemic/Hypoxic Disease Institute, Seoul National University Medical Research Center, Seoul 03080, Republic of Korea

## Abstract

Cells need to overcome both intrinsic and extrinsic threats. Although pluripotency is associated with damage responses, how stem cells respond to DNA damage remains controversial. Here, we elucidate that DNA damage activates Chk2, leading to the phosphorylation of serine 164 on C-terminal binding protein 2 (Ctbp2). The phosphorylation of Ctbp2 induces the disruption of Ctbp2 tetramer, weakening interactions with zinc finger proteins, leading to the dissociation of phosphorylated Ctbp2 from chromatin. This transition to a monomeric state results in the separation of histone deacetylase 1 from Ctbp2, consequently slowing the rate of H3K27 deacetylation. In contrast to the nucleosome remodeling and deacetylase complex, phosphorylated Ctbp2 increased binding affinity to polycomb repressive complex (PRC)2, interacting through the N-terminal domain of Suz12. Through this domain, Ctbp2 competes with Jarid2, inhibiting the function of PRC2. Thus, the phosphorylation of Ctbp2 under stress conditions represents a precise mechanism aimed at preserving stemness traits by inhibiting permanent transcriptional shutdown.

## Graphical Abstract

**Graphical Abstract:**
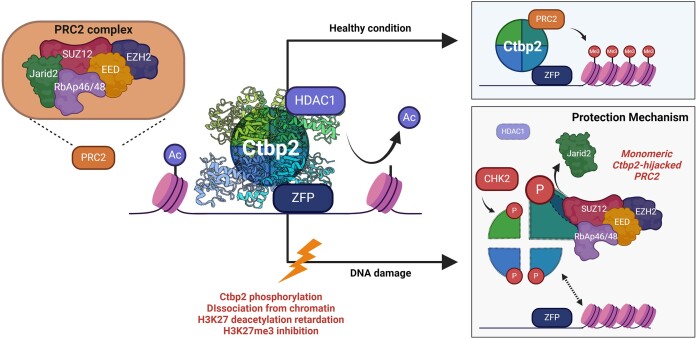

## Introduction

Embryonic stem cells (ESCs) are at the highest level of stem cell hierarchy and can differentiate into all three types of germ layers. ESCs self-renew to perpetuate their pluripotent characteristics until they are stimulated by differentiation signals. Based on these distinct properties, mutations derived from DNA damage in ESCs are extensively propagated, ultimately leading to compromised stem cell function (1). To compensate for deleterious DNA lesions, ESCs tend to operate enhanced DNA damage repair mechanisms (2,3). Despite the robust repair system, when threatened with severe damage, ESCs differentiate to prevent propagation of anomalous mutations. DNA damage induced p53 and long non-coding RNAs repressed pluripotency-related genes and conversely activated differentiation-related genes (4–8). However, prolonged exposure to DNA damage and continuous differentiation signals deplete the ESC pool. Thus, DNA damage is tightly connected with pluripotency control. However, depending on the circumstances, such as species or cell cycle state, the linkage between DNA damage and pluripotency control exhibits contrasting phenotypes (9). A detailed understanding of how ESCs maintain the delicate balance between preserving pluripotency and initiating differentiation when exposed to DNA damage remains elusive.

Regarding the spatiotemporal orchestration of gene expression, epigenetic modifiers are the fundamental modules that immediately respond to a new environmental situation (10). Nucleosome remodeling and deacetylase complex (NuRD) and polycomb repressive complex (PRC) are important epigenetic enzymes for maintaining stemness and differentiation (11–13). Recently, the connection between epigenetic regulation, including histone acetylation and methylation, and the DNA damage response was reported (14,15). Epigenetic modifications under DNA damage conditions play a crucial role in damage sensing, recognizing the damage site and triggering a cascade of signaling pathways that activate the DNA damage response (16). Once DNA damage occurs, histone deacetylases (HDACs) are recruited to the damage site, where they trigger deacetylation to suppress transcription, which promotes repair mechanisms. For instance, HDAC1 and HDAC2 are involved in the DNA damage response by promoting DNA non-homologous end-joining (17). Additionally, Poly (ADP-ribose) polymerase recruits the PRC and NuRD complexes to the damage site, stimulating H3K27me3 to facilitate repair (18), Ezh2, a catalytic component of PRC2, localized to the damage site and catalyzed the formation of H3K27me3 (18,19). DNA double-strand breaks attract DNA methyltransferase, SIRT1 and Ezh2 to the site (20). The diverse epigenetic changes are essential for an effective response to DNA damage. These epigenetic changes help cells recognize the threat and activate response pathways (21). Therefore, epigenetic changes in under damaging conditions are indispensable. However, their precise role is controversial based on the context. In many cases, the inhibition of NuRD or PRC2 showed altered signaling pathways in either supportive or antagonistic ways (22–25).

C-terminal binding protein 2 (Ctbp2) is a transcriptional corepressor that is highly transcribed by the core pluripotency factors Oct4, Sox2, Klf4 and Nanog in proportion to pluripotent states (26). The deletion of Ctbp2 delayed differentiation at the cellular level and caused embryonic lethality at the animal level, suggesting that Ctbp2 is related to pluripotency and differentiation ability (27,28). Indeed, a recent study has revealed that Ctbp2 pre-emptively occupies pluripotency-related gene regions with NuRD and PRC2, which trigger epigenetic silencing upon stimulation from differentiation cues (26). Ctbp2 sequesters the β-catenin destruction complex (APC/GSK3β/AXIN1/CK1α) in an undifferentiated state and induces the destabilization of β-catenin, which is irreplaceable for lineage determination (29). Collectively, abundant Ctbp2 levels and proper priming occupancy are prerequisites for conferring differentiation potential to ESCs (30). In terms of its role in DNA damage response, Ctbp2 is observed not only in pluripotency genes but also in DNA damage response genes (31). Loss of Ctbp2 makes cells more vulnerable to DNA damage (32). However, the connecting role of Ctbp2 in pluripotency control and DNA damage response is unclear.

## Materials and methods

### Cell culture

Mouse ESCs (mESCs; E14) were obtained from Mutant Mouse Resource and Research Center and cultured on 0.1% gelatin-coated plates. mESC media contained Dulbecco’s modified Eagle’s medium (Corning, Manassas, VA, USA) supplemented with 15% (v/v) FBS (Gibco, Grand Island, NY, USA), 2 mM L-glutamine, 1% (v/v) Minimum Essential Medium (MEM) non-essential amino acids, 100 U/ml penicillin, 100 μg/ml streptomycin, 55 μM β-mercaptoethanol (all from Gibco, Grand Island, NY, USA) and 1000 U/ml ESGRO leukemia inhibitory factor (MilliporeSigma, Darmstadt, Germany). Differentiation was induced by removing leukemia inhibitory factor from complete medium. Human embryonic kidney 293T and 293FT cell lines were acquired from American Type Culture Collection and cultured in Dulbecco’s modified Eagle’s medium with 10% (v/v) FBS, 100 U/ml penicillin and 100 μg/ml streptomycin. For 293FT medium, 2 mM L-glutamine, 1% (v/v) MEM non-essential amino acids and 1% (v/v) sodium pyruvate were added.

### Plasmids

The pCAG-Ctbp2 construct was cloned, as described (26). The S106E-, S164E-, S424E- and S428E-Ctbp2 constructs were generated by site-directed mutagenesis. The primers for site-directed mutagenesis are listed in Supplementary Table S1. For bacterial recombinant protein expression, wild-type Ctbp2 and S164E-Ctbp2 were cloned into the pGEX-4T-1, pRSET-B or pET21a-Flag vector. To generate baculovirus vectors of human PRC2 components, FLAG-Eed, -Suz12, -Ezh2, -RbAp48 and -Jarid2 were individually cloned into the pFastBac1 plasmid (Invitrogen, 10360014). All constructs were verified by Sanger DNA sequencing.

### Antibodies

We used the following antibodies: Anti-Ctbp2 (BD Biosciences, 612044, Active Motif, 61261), Anti-Actin (Sigma-Aldrich, A5441), Anti-HA (Covance, MMS-101R), Anti-FLAG (Sigma-Aldrich, F3165), Anti-phospho-Ctbp2 S164 (GenScript), Anti-Chk2 (Sigma-Aldrich, 05-649), Anti-Ctbp1 (BD Biosciences, 612 042), Anti-Oct3/4 (Santa Cruz Biotechnology, sc-5279), Anti-Sox2 (R&D Systems, MAB2018), Anti-Nanog (Abcam, ab14959), Anti-Myc (BioLegend, 9E10), Anti-Glutathione S-transferase (GST) (GenScript), Anti-His (Cell Signaling Technology, 2365S), Anti-Histone H3 (Cell Signaling Technology, 9715S), Anti-Zfp217 (GenScript), Anti-Hic2 (GenScript), Anti-Hdac1 (Abcam, ab7028), Anti-H3K27Ac (Abcam, ab177178), Anti-Lsd1 (Abcam, ab17721), Anti-Ezh2 (BD Biosciences, 612666, Active Motif, 39875), Anti-Suz12 (Cell Signaling Technology, 3737S), Anti-Eed (Cell Signaling Technology, 85322) and Anti-H3K27me3 (Active Motif, 91403).

### Computational prediction of phosphorylation sites

To predict phosphorylation sites, group-based prediction system version 5.0 (GPS 5.0) was used with a medium threshold (33).

### CRISPR/Cas9-mediated gene knockout and editing

For knockout, two single guide RNAs targeting exon 4 of *Ctbp2* were designed using the Clustered Regularly Interspaced Short Palindromic Repeats / CRISPR-associated protein 9 (CRISPR/Cas9) Design Tool. The single guide RNAs were annealed, phosphorylated and ligated into the pSpCas9n (BB)-2A-GFP (PX461) vector. The vectors were transfected into E14 ESCs using Lipofectamine 3000 reagent (Invitrogen, #L3000015). Then, 24 h after transfection, GFP-positive cells were isolated using BD cell sorter Aria II (BD Biosciences). Each clone was screened using western blot analysis. Subsequently, genomic DNA was isolated and amplified to determine the deleted sequence. For genomic editing, a single guide RNA targeting the nucleotide sequence of serine 164 was designed and cloned into the pSpCas9 (BB)-2A-GFP (PX458) vector. Single strand oligonucleotides were generated to induce homology-directed repair. The vector and single strand oligonucleotides were transfected into E14 ESCs using Lipofectamine 3000 reagent. Then, 24 h after transfection, GFP-positive cells were isolated using BD cell sorter Aria II. Genomic DNA from each clone was isolated and amplified to evaluate whether the target sequence was edited. The single guide RNA sequences for knockout or knock-in, along with the HDR template, are listed in Supplementary Table S1.

### Immunoprecipitation assay

Transiently transfected or stably expressing cells were lysed (25 mM Tris-Cl [pH 7.5], 0.1% [v/v] NP-40, 150 mM NaCl, 10% glycerol, protease inhibitors and phosphatase inhibitors) with brief sonication. Lysates were incubated with the indicated antibodies at 4°C overnight. Protein complexes were incubated with protein A- or protein G-agarose at 4°C for 2 h and washed three times with lysis buffer. IP samples were separated with sodium dodecyl sulfate-polyacrylamide gel electrophoresis (SDS-PAGE) and probed with the indicated antibodies.

### *In vitro* phosphorylation assay

The *in vitro* phosphorylation assay was performed using the indicated kinases and recombinant His_6_-Ctbp2 in the kinase buffer (final concentration: 50 mM HEPES-OH [pH 8.0], 10 mM MgCl_2_, 10 mM MnCl_2_, 1 mM DTT, 500 nM ATP and 3 μM sodium orthovanadate). For AMP-activated protein kinase, 0.1 mM AMP was added. For Ca^2+^/calmodulin-dependent protein kinase 2, 0.5 mM CaCl_2_ and 1 μM calmodulin were added. The sample was incubated at 30°C for 30 min, and the reaction was stopped by boiling for 5 min with sample buffer. To visualize the bands, the samples were separated using SDS-PAGE. The signals were detected using a phosphorylation-specific antibody.

### Alkaline phosphatase staining

E14 ESCs were plated at a density of 500 cells per well in 0.1% gelatin-coated 6-well plates. Cells were incubated for 6 d in a complete medium. Differentiation was induced by removal of leukemia inhibitory factor from complete medium. The medium was changed every day. After the incubation, colonies were stained for alkaline phosphatase using a staining buffer composed of 0.8 mg/ml fast red violet solution in distilled water and 4 mg/ml naphthol AS-BI phosphate solution in 2 M 2-amino-2-methyl-1-propanol (AMPD) buffer (pH 9.5).

### Protein purification

GST-, Flag- or His_6_-tagged Ctbp2 was expressed in *Escherichia coli* (BL21 DE3 pLysS). *E. coli* was transformed with pGEX-4T-1, pET21a-Flag or pREST-B Ctbp2. Protein expression was induced with 0.5 mM Isopropyl β-D-1-thiogalactopyranoside (IPTG) when the culture reached an optical density of 0.6 at 600 nm. Induced bacterial cells were cultured at 37°C for 4 h; harvested; suspended in 10 mM Tris-Cl (pH 7.4), 160 mM NaCl, 1 mM EDTA, 1 mM Phenylmethylsulfonyl fluoride (PMSF) and 1 mg/ml lysozyme, and incubated at 4°C for 30 min. Triton X-100 (final concentration of 1%) was added to the suspension and bacteria were lysed by sonication, followed by incubation at 4°C for 30 min. The lysate was centrifuged at 15 000 rpm for 15 min and the supernatant was mixed with glutathione Sepharose 4B (GE Healthcare), Flag M2 agarose bead (Sigma) or Ni-NTA agarose beads (QIAGEN) at 4°C for 1 h with gentle rotation. The beads were washed three times with suspension buffer. For His_6_-tagged protein, 10 mM imidazole was added. Elution was conducted with elution buffer composed of 100 mM Tris-Cl (pH 8.0), 20 mM reduced glutathione, 160 mM NaCl and 10% glycerol for GST-tagged proteins, 50 mM HEPES (pH 7.5), 0.5 mg/ml 3xFlag peptide and 10% glycerol for Flag-tagged proteins or 100 mM Tris-Cl (pH 7.4), 150 mM imidazole, 160 mM NaCl and 10% glycerol for His_6_-tagged proteins. Final buffer was changed to 50 mM HEPES-OH (pH 8.0) and 10% glycerol using 10 kDa Amicon Centricon (Merck Millipore). Four PRC2 components (FLAG-Eed, -Suz12, -Ezh2 and -RbAp48) were coexpressed in Sf9 cells through baculovirus infection. Then, 60 h post-infection, the cells were harvested and resuspended in BC350 buffer (20 mM HEPES-NaCl [pH 7.8], 350 mM NaCl, 10% glycerol, 0.1% NP-40 and 1 mM EDTA) containing protease inhibitors (1 mM PMSF, 1 mM benzamidine and 1 mM leupeptin) and phosphatase inhibitors (10 mM NaF and 1 mM Na_3_VO_4_). The cells were lysed by sonication (Sonics Vibra Cell VCX-130), and recombinant PRC2 was purified using Flag M2 agarose beads (Sigma) and Q Sepharose beads (Cytiva). The purified PRC2 complexes were dialyzed into BC100 buffer (20 mM HEPES-NaCl [pH 7.8], 100 mM NaCl, 10% glycerol, 0.1% NP-40 and 1 mM EDTA) for *in vitro* assays. The concentrations of the purified PRC2 complexes were determined by comparing to a bovineserum albumin (BSA) standard using Coomassie staining.

### *In vitro* binding assay

In total, 1 μg of His_6_-tagged Ctbp2 and 1 μg of GST-tagged Ctbp2 or 1 μg of FLAG-PRC2 were mixed in 25 mM Tris-Cl (pH 7.5), 0.1% (v/v) NP-40, 150 mM NaCl, 1 μmol nicotinamideadenine dinucleotide (NADH), 1 μg BSA and 10% glycerol at 4°C for 1 h; incubated with pre-equilibrated glutathione Sepharose 4B or FLAG M2 beads at 4°C for 4 h; washed three times washes; and boiled for 5 min.

### Differential scanning fluorimetry

Differential scanning fluorimetry was performed using the CFX Connect Real-Time PCR system with CFX Maestro software. A total of 2 μg of either wild-type or S164E recombinant Ctbp2 protein was mixed with 5x SYPRO Orange protein gel stain (Invitrogen), along with 100 μM NADH or 10 mM HEPES-OH [pH 7.5] buffer containing 137 mM NaCl, 2.7 mM KCl, 10 mM Na_2_HPO_4_ and 1.8 mM KH_2_PO_4_ [pH 7.4]. The reaction was conducted by gradually increasing the temperature from 20 to 90°C at a rate of 1°C per minute. The melting temperatures were determined by derivative analysis.

### Chromatin immunoprecipitation assay

E14 mESCs were fixed with formaldehyde (final concentration: 1%) for 10 min and quenched using 125 mM glycine. The cells were lysed with buffer A (5 mM PIPES [pH 8.0], 85 mM KCl and 0.5% [v/v] NP-40). Nuclei were collected by centrifugation and lysed in buffer B (50 mM HEPES [pH 7.5], 140 mM NaCl, 1 mM EDTA, 1% [v/v] Triton X-100, 0.1% [v/v] Sodium deoxycholate (SDC) and 0.1% [v/v] SDS) on ice for 10 min. Subsequently, chromatin was sheared using a Bioruptor sonicator (Diagenode). Lysates were clarified and quantified with a bioanalyzer. Then 100 μg of lysate was diluted 10-fold in chromatin immunoprecipitation (ChIP) buffer (16.7 mM Tris-Cl [pH 8.1], 167 mM NaCl, 1.1% [v/v] Triton X-100, 0.01% [w/v] SDS and 1.2 mM EDTA) with the indicated antibody at 4°C overnight; incubated with Protein A or G-agarose beads for 4 h; washed once with TSE150 (20 mM Tris-Cl [pH 8.1], 150 mM NaCl, 1% Triton X-100, 0.1% SDS and 2 mM EDTA); washed once with TSE500 (20 mM Tris-Cl [pH 8.1], 500 mM NaCl, 1% Triton X-100, 0.1% SDS and 2 mM EDTA); washed once with buffer 3 (10 mM Tris-Cl [pH 8.1], 0.25 M LiCl, 1% NP40, 1% deoxycholate and 1 mM EDTA); and washed twice with TE (10 mM Tris-Cl [pH 8.0] and 0.5 mM EDTA). Chromatin was eluted with 500 μl of elution buffer (0.1 M NaHCO_3_ and 1% SDS) and the sample was decrosslinked at 70°C with 0.2 M NaCl overnight. Separated proteins were removed using proteinase K, and DNA was purified using glycogen.

### Chromatin immunoprecipitation sequencing analysis

Library for sequencing was generated using TruSeq ChIP Library Preparation Kit (Illumina, IP-202-1012). High-throughput sequencing was conducted using NextSeq 500 System (Illumina, SY-415-1002). The quality of raw data was checked (FastQC, version 0.11.9). Adapter sequences were trimmed (Cutadapt, version 4.0) (34) and aligned to the mouse reference genome (mm9) (Bowtie2, version 2.5.0) (35) with default settings. Output datasets were sorted by coordinate order (SortSam, version 2.18.2). Duplicated reads were identified and removed (MarkDuplicates, version 2.18.2). Narrow peaks of each alignment result were called with a q-value cutoff of 0.05 (MACS2, version 2.2.7.1) (36). Histone broad peak regions were detected from bedGraph output generated using MACS2 with default option (cutoff 2.0, minimum length 200, maximum gap 30 and maximum liking 800; MACS2, version 2.2.7.1). To find overlapping regions, peak files were compared (bedtools Intersect intervals, version 2.30.0) and annotated (ChIPseeker, version 1.18.0). Gene ontology was analyzed using Database for Annotation, Visualization, and Integrated Discovery (37), and processed using R. Peak values were calculated (computeMatrix, version 3.5.1) and visualized as a plot-heatmap (plotHeatmap, version 3.5.1) with Integrative Genomics Viewer (IGV) tool (version 2.4.10).

### Statistical analysis

Comparison of statistical significance between two groups was performed using Student’s *t*-test. To analyze multiple groups, one-way Analysis of Variance (ANOVA) with Tukey’s multiple comparison test was conducted using GraphPad Prism 7 software (GraphPad Software Inc., La Jolla, CA). Data are presented as mean ± standard deviation. The number of experimental replicates of experimental conditions is given in figure legends. The western blot analysis was performed in more than three independent experiments. The primer sequences for Chromatin Immunoprecipitation followed by quantitative Polymerase Chain Reaction (ChIP-qPCR) are listed in Supplementary Table S1. Significant differences were indicated as *P*-values: *P < 0.05, **P < 0.01 and ***P < 0.001.

## Results

### Serine 164 is important for Ctbp2 function

**Figure 1.**
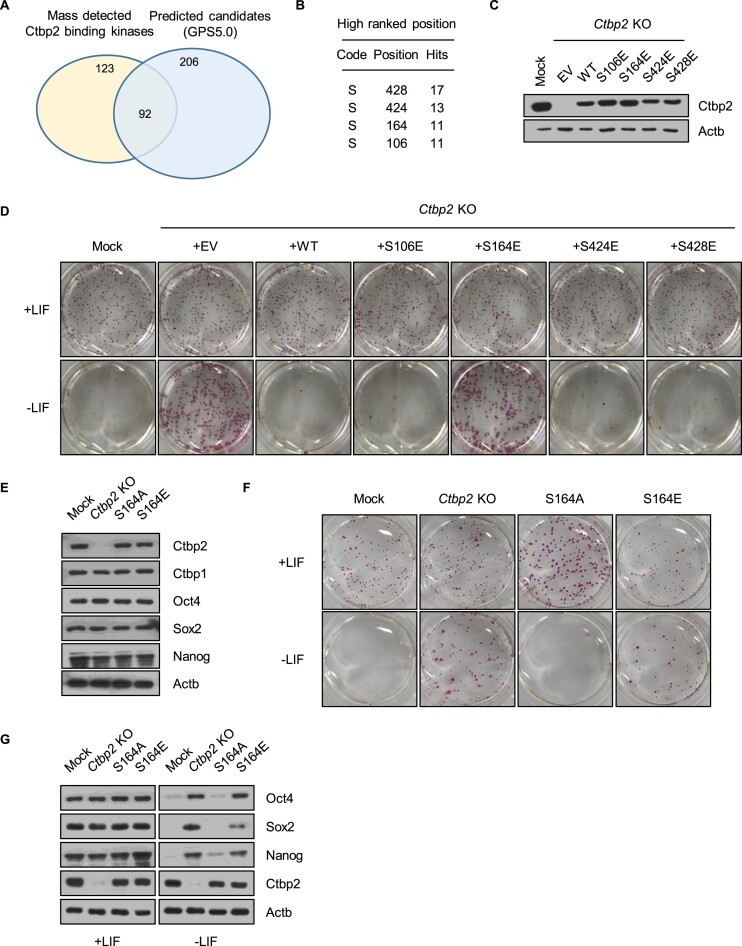
Serine 164 plays an important role in Ctbp2 function. (**A**) A Venn diagram comparing computational prediction data, generated using GPS 5.0 with a medium threshold, and complex mass spectrometry data. (**B**) Top four potentially phosphorylated residues. Code refers to the amino acid, where S represents serine. Position indicates the amino acid number, and Hits represents the number of times the residue was predicted as phosphorylated in the computational prediction. (**C**) Western blot images of wild-type or phosphomimetic mutant Ctbp2 expressed in a Ctbp2 knockout cell line. (**D**) Alkaline phosphatase staining. Differentiation was induced by removing LIF for 6 d. (**E**) Western blot analysis of Ctbp and pluripotency genes in Ctbp2 knock-in mouse ESCs, using the indicated antibodies. (**F**) Alkaline phosphatase staining of wild-type and edited cell lines in a pluripotent state. (**G**) Western blot images of pluripotency genes. Differentiation was induced by removing Leukemia Inhibitory Factor (LIF) for 4 d.

Ctbp2 is essential for regulating exit from pluripotency by repressing pluripotency genes during differentiation (26). However, the upstream modulators that regulate its function in ESCs are unclear. We aimed to identify kinases that modulate Ctbp2 function in ESCs. Therefore, we used computational phosphorylation prediction using the GPS 5.0 program (33) and identified 298 possible candidates that phosphorylate Ctbp2. Previously we obtained a list of Ctbp2-interacting proteins in ESCs (26). We compared these two lists and identified 92 common kinases. (Figure 1A). Next, we selected high-ranking residues and identified serine 428, 424, 164 and 106 (Figure 1B). To validate whether these residues alter Ctbp2 function, we generated Ctbp2 knockout (KO) ESCs using the CRISPR/Cas9 system (Supplementary Figure S1). When Ctbp2 was knocked out, ESCs exhibited a delayed differentiation phenotype (26,27). Wild-type or phosphomimetic serine to glutamic acid mutations at 428, 424 and 106 rescued the differentiation phenotype. However, the phosphomimetic mutation at 164 did not rescue the differentiation phenotype, and the colonies were positive for alkaline phosphatase staining (Figure 1C and D). To determine the physiological function of phosphorylated serine 164 in ESCs, we edited the selected residue at the genomic level to generate the nonphospho- and phosphomimetic forms, S164A and S164E, respectively, using the CRISPR/Cas9 system (Supplementary Figure S2). Both substitutions did not disturb the expression of pluripotency genes, such as Oct4, Sox2 and Nanog, in the pluripotent state (Figure 1E). Nevertheless, phosphomimetic Ctbp2, but not non-phosphorylated Ctbp2 lost its ability to exit from pluripotency (Figure 1F and G). These results indicate that the phosphorylation of serine 164 has an important role in Ctbp2 function to silence pluripotency but does not directly modulate maintaining stemness in pluripotent state.

### AMPK, CHK and CaMK2 phosphorylate serine 164 *in vitro*

Next, we examined which kinase phosphorylates serine 164 in Ctbp2. According to our predictions and Ctbp2 complex data, 11 kinases (PRKAA1, RPS6KA1, CHK1, CHK2, BUB1, CAMK2B, CAMK2G, CAMK2D, STK38, EIF2AK3 and SRPK1) interact with Ctbp2 in ESCs and can phosphorylate serine 164 (Supplementary Figure S3A). To verify their interactions, each kinase was cotransfected with Ctbp2 into HEK293T cells. The immunoprecipitation (IP) assay showed that all candidates, except EIF2AK3 (not cloned) and SRPK1, could bind Ctbp2 at cellular levels (Supplementary Figure S3B). Therefore, we tested whether the kinases could phosphorylate Ctbp2. Customized anti-phosphorylated serine164 Ctbp2 antibody was designed to clarify if phosphorylation occurred at serine 164 (Supplementary Figure S3C). The *in vitro* phosphorylation assay confirmed that AMP-activated protein kinase (AMPK), Checkpoint kinase 1 (Chk1), Chk2, Calcium/Calmodulin-dependent protein kinase 2B (CaMK2B), CaMK2G and CaMK2D phosphorylated serine 164 of Ctbp2 *in vitro* (Supplementary Figure S3D). However, the IP assay revealed that Chk2, but not AMPK and CaMK2, phosphorylates Ctbp2 at the cellular level (Supplementary Figure S3E). A serine to alanine substitution confirmed that Chk2 phosphorylates Ctbp2 at serine 164 (Supplementary Figure S3F). Thus, Chk2 is a key kinase responsible for phosphorylating Ctbp2.

### Chk2 phosphorylates serine 164 of Ctbp2

**Figure 2.**
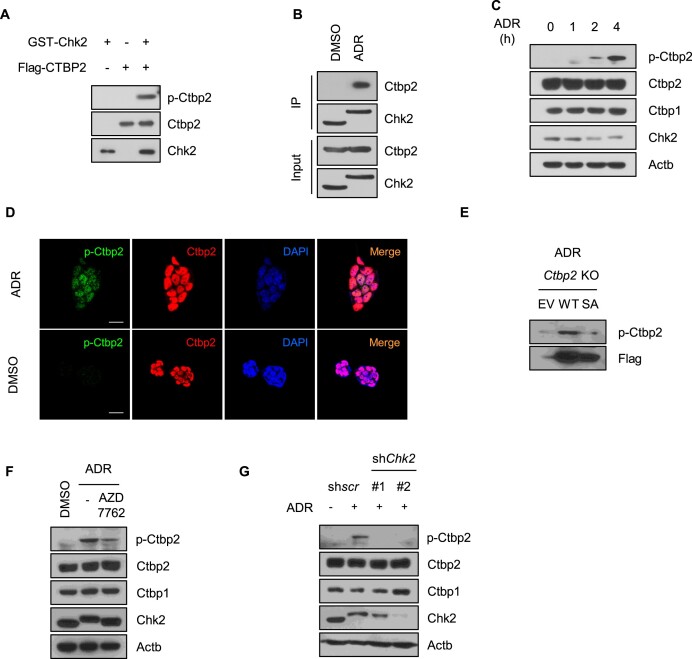
Chk2 phosphorylates serine 164 of Ctbp2 under damage conditions. (**A**) *In vitro* phosphorylation assay. Here, 50 ng of GST-Chk2 and 500 ng of FLAG-CTBP2 were incubated for 30 min. Immunoblotting was conducted using the indicated antibodies. (**B**) Immunoprecipitation of Ctbp2 and Chk2 in mESCs (E14) treated with 500 nM ADR for 4 h to activate Chk2, using anti-Chk2 antibodies. (**C**) Western blot images showing phosphorylation levels of Ctbp2 after 500 nM ADR treatment for the indicated duration. (**D**) Confocal microscopy images of phosphorylated Ctbp2 or total Ctbp2 in E14 cells; nuclei are stained with 4′,6-diamidino-2-phenylindole (DAPI). Scale bar: 20 μm. (**E**) Western blot images of phosphorylated Ctbp2. ADR was treated at wild-type or S164A Ctbp2 rescued in Ctbp2 knockout stem cell lines. (**F**) Pretreatment with 10 μM of the Chk2 inhibitor AZD7762 for 2 h prior to ADR treatment in stem cells. (**G**) Lentiviral knockdown of Chk2 in E14 mouse ESCs. shScr was used as a negative control.

Chk2 is a representative enzyme activated in response to DNA damage (38). First, we conducted an *in vitro* phosphorylation assay using recombinant active Chk2 and Ctbp2. Consistent with the IP phosphorylation assay results, Chk2 effectively phosphorylated Ctbp2 at serine 164 (Figure 2A). To stimulate endogenous Chk2, DNA damage was induced by adriamycin (ADR) or etoposide treatment (Supplementary Figure S4). Upon DNA damage, Chk2 bound to Ctbp2 in ESCs (Figure 2B), which accumulated phosphorylated Ctbp2; however, Ctbp2 KO or S164A Ctbp2 ESCs did not accumulate phosphorylated Ctbp2 in a Chk2-activation dependent manner (Figure 2C–E). Pretreatment with AZD7762, a Chk2 inhibitor, diminished phosphorylation levels in proportion to reduced Chk2 activity, demonstrating that Ctbp2 phosphorylation is contingent upon Chk2 activity (Figure 2F). Consistently, lentiviral knockdown of *Chk2* reduced phosphorylation levels (Figure 2G). Taken together, Chk2 phosphorylates Ctbp2 at serine 164 upon DNA damage stress in ESCs.

### Phosphorylation of serine 164 disrupts the Ctbp2 tetramer

**Figure 3.**
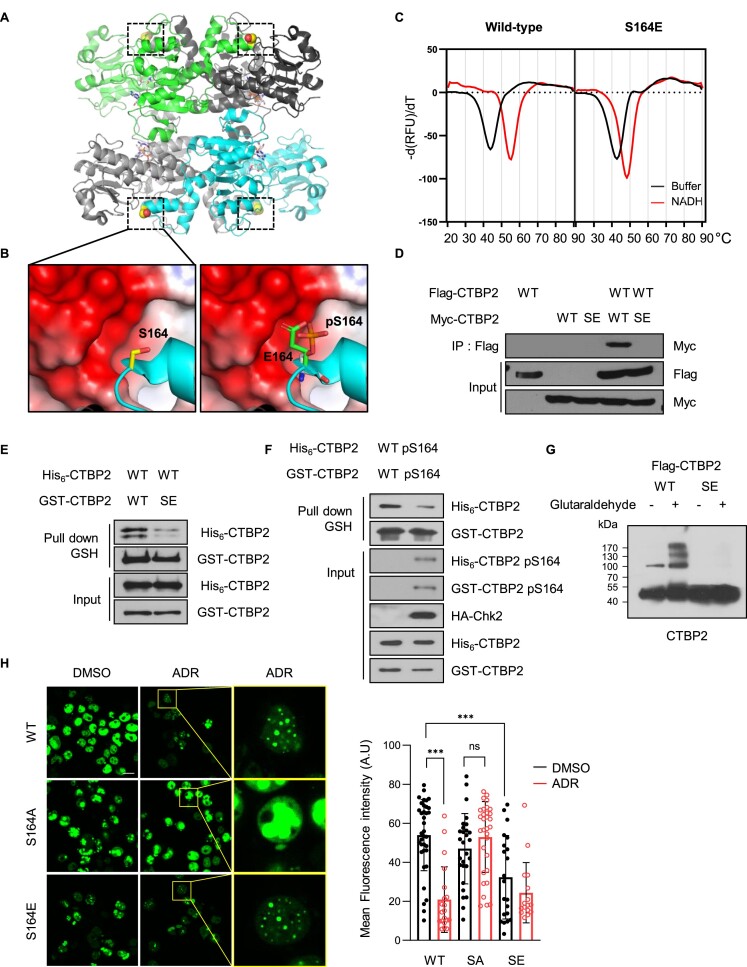
Disruption of Ctbp2 tetramerization upon DNA damage. (**A**) Tetrameric structure of the Ctbp2/NAD complex (PDB codes: 6WKW and 2OME) (39). Each monomer is shown in a different color. The location of S164 (yellow spheres) is indicated by dashed squares in each monomer. (**B**) Close-up view of S164 (left), phosphorylated S164 (pS164; right) and E164 (right) and their binding interface from another monomer. For brevity, only pS164 and E164 are displayed from the *in silico* models of phosphorylated Ctbp2 and the phosphomimetic mutant, respectively, while the wild-type structure is shown. The binding interface is represented as a surface with electrostatic potentials: positive (blue), negative (red) and neutral (white). Nitrogen and oxygen atoms in all spheres and sticks are colored in blue and red, respectively. (**C**) Differential scanning fluorimetry was conducted with 100 μM NADH or 10 mM HEPES-OH (pH 7.5) buffer containing 137 mM NaCl, 2.7 mM KCl, 10 mM Na_2_HPO_4_ and 1.8 mM KH_2_PO_4_ (pH 7.4). (**D**) Binding of FLAG- or Myc-tagged CTBP2 transfected into HEK293T cells and immunoprecipitated with anti-FLAG antibodies. (**E**) *In vitro* binding of 1 μg GST- or His_6_-tagged recombinant CTBP2 and pulled down with GSH beads. (**F**) In order to purify Chk2, HA-Chk2 was transfected into HEK293T and subsequently immunoprecipitated. *In vitro* phosphorylation was performed in kinase buffer composition. Phosphorylation of Ctbp2 at S164 was detected using a phospho-specific anti-Ctbp2 S164 antibody. The pulldown assay was conducted using GSH. (**G**) *In vitro* crosslinking assay. Wild-type or S164E FLAG-CTBP2 recombinant proteins were crosslinked with glutaraldehyde. (**H**) Bimolecular fluorescence complementation assay of Ctbp2. HEK293T cells were transfected with Venus N or Venus C tagged CTBP2 and treated with 500 nM ADR for 4 h. Fluorescence images were validated by confocal microscopy and quantified using ImageJ (*P < 0.05). Scale bar indicates 15 μm.

We tested the main mechanism by which phosphorylated Ctbp2 loses its activity. Ctbp2 is a tetramer, and Ctbp2 disassembly decreases its function (39,40). Because serine 164 residue is on the surface of the Ctbp2 tetramer and close to the NADH-binding site, which is an important cofactor for Ctbp2 assembly (41) (Figure 3A), we tested if the phosphorylation of serine 164 affects Ctbp2 tetramerization. To obtain a structural basis for the disruption of tetrameric Ctbp2, the structure of wild-type Ctpb2 (PDB codes: 6WKW and 2OME) (39) was compared with *in silico* models of Ctbp2 containing phosphorylated Ser164 (pS164) or the phosphomimetic mutation (S164E), predicted by AlphaFold 3 (42). Ser164 in one monomer fits into the shallow groove of another monomer, indicating that Ser164 primarily contributes to the tetramerization of Ctbp2 (Figure 3A and B). Since the shallow groove contains a negatively charged region (Figure 3B, left), it favors electrostatic interactions with a positively charged residue in the opposing monomer. In contrast, phosphorylation or the Ser to Glu substitution at position 164 introduces a stronger negative charge than serine (Figure 3B, right), likely resulting in electrostatic repulsion at the binding interface. Therefore, pS164 or S164E seems to destabilize the interaction between monomers, thereby disrupting the tetrameric state of Ctbp2. Next, a differential scanning fluorimetry assay was performed to evaluate the NADH-binding capacity. NADH is known to enhance Ctbp2 tetramerization, thereby increasing its thermostability (43). The melting temperature of wild-type Ctbp2 shifted from 44°C to 55°C in the presence of NADH, whereas the S164E mutant exhibited a shift to 48°C. This shift suggests that phosphorylation at S164 reduces the NADH-binding ability of Ctbp2 (Figure 3C). Therefore, the phosphorylation has a possibility to modulate Ctbp2’s tetramerization. The IP assay showed that wild-type Ctbp2 bound to wild-type Ctbp2, but the S164E mutation markedly abolished this binding ability (Figure 3D). The *in vitro* binding assay using recombinant wild-type and S164E proteins confirmed a reduction in tetramer formation (Figure 3E). To assess whether phosphorylation has the same effect as the S164E substitution, GST- or His_6_-tagged recombinant Ctbp2 proteins were phosphorylated using HA-Chk2, followed by an *in vitro* binding assay. Consistent with the S164E results, phosphorylation at S164 also reduced tetramer formation (Figure 3F). The *in vitro* chemical crosslinking assay exhibited that S164E Ctbp2 fails to tetramerize (Figure 3G). The bimolecular fluorescence complementation assay demonstrated that ADR-induced phosphorylation or S164E substitution disrupted Ctbp2 tetramer assembly at the cellular level, whereas the S164A mutation impaired this effect (Figure 3H). These results indicated that phosphorylation under DNA damage conditions disrupted Ctbp2 tetramerization.

### DNA damage dissociates Ctbp2 from chromatin

**Figure 4.**
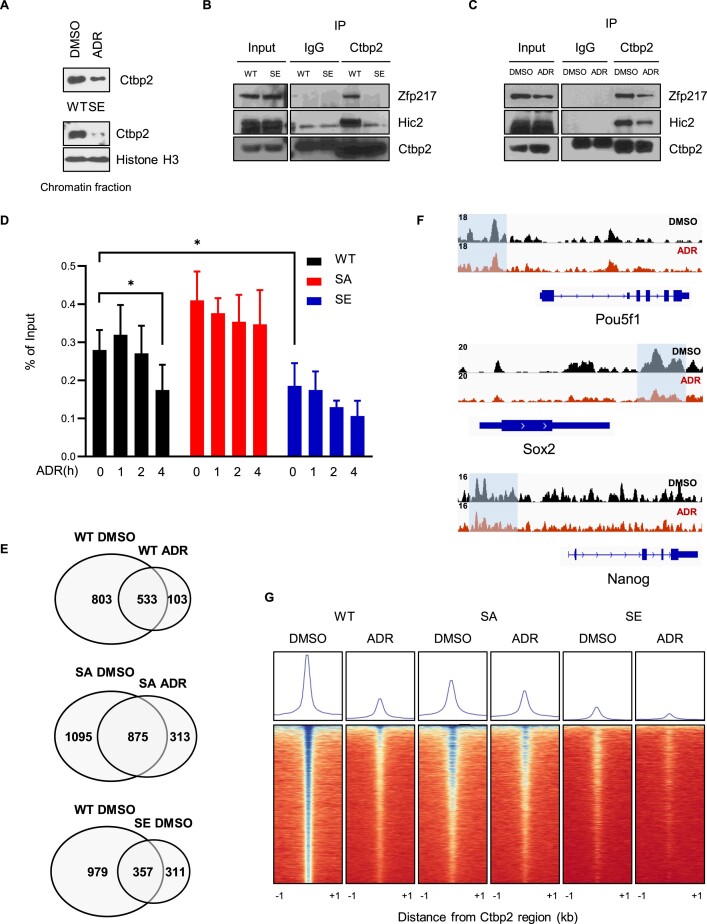
DNA damage dissociates Ctbp2 from chromatin. (**A**) Subcellular fractionation showing chromatin fractions with or without 500 nM ADR treatment for 4 h in mESCs. Comparisons were made between wild-type and S164E Ctbp2 knock-in (KI) mESCs. Histone H3 served as a chromatin fraction marker. (**B**) Immunoprecipitation assay in wild-type and S164E KI mESCs with ZFPs. IP was conducted using anti-Ctbp2 antibodies. (**C**) Immunoprecipitation assay with 500 nM ADR for 4 h in mESCs with ZFPs. IP was conducted using anti-Ctbp2 antibodies. (**D**) ChIP assay of Ctbp2 performed after 500 nM ADR treatment targeting the Oct4 enhancer region (*n* = 5, *P < 0.05). (**E**) Peak calling comparison using a cutoff for peaks of 10, a minimum peak length of 200 and a maximum gap between significant points in a peak of 30. (**F**) An IGV snapshot representing Ctbp2 ChIP-sequencing results visualized for the Oct4, Sox2 and Nanog gene regions. (**G**) Heatmap plot of global Ctbp2 distribution, comparing the presence of Ctbp2 peaks with and without 500 nM ADR treatment for 4 h in wild-type or S164A Ctbp2 KI or S164E Ctbp2 KI mESCs.

Consistent with our finding that phosphorylated Ctbp2 monomerized, a study reported that the destabilization of the Ctbp2 tetramer impeded its transcriptional regulatory functions (39). Therefore, we aimed to investigate how this transition attenuates Ctbp2 function. We compared Ctbp2 localization under DNA damage conditions. Subcellular fractionation showed that a phosphomimetic mutation or ADR treatment induced the dissociation of Ctbp2 from chromatin (Figure 4A). According to a study, Ctbp2 cooperates with zinc finger proteins (ZFPs) to bind target loci because it lacks a DNA-binding motif (44). Indeed, IP experiments with a phosphomimetic mutant or ADR treatment exhibited that DNA damage-triggered phosphorylation reduced the binding affinity of Ctbp2 to ZFPs, such as Zfp217 and Hic2 (Figure 4B and C). Consistently, DNA damage dissociated Ctbp2 from chromatin in a phosphorylation-dependent manner and the nonphosphorylated mutation inhibited this effect (Figure 4D). To validate the global pattern of Ctbp2 detachment, ChIP-sequencing was performed, identifying 1336 peaks in wild-type cells treated with DMSO, 636 peaks in wild-type cells treated with ADR, 1970 peaks in SA cells treated with dimethyl sulfoxide (DMSO), 1188 peaks in SA cells treated with ADR, 668 peaks in SE cells treated with DMSO and 800 peaks in SE cells treated with ADR. A 47.6% reduction in Ctbp2 peaks was observed in wild-type cells following ADR treatment. Consistent with this observation, SE cells exhibited only 668 Ctbp2 peaks even under DMSO conditions. In contrast, SA cells showed a higher number of peaks (1970) and retained 60.3% of them after DNA damage (Figure 4E). Representative pluripotency genes targeted by Ctbp2 also demonstrated Ctbp2 detachment (Figure 4F). These findings are further supported by plot profile and heatmap analyses, which revealed a phosphorylation-dependent pattern of Ctbp2 detachment (Figure 4G). Collectively, DNA damage disrupts the Ctbp2 tetramer and detaches it from chromatin.

### Phosphorylated Ctbp2 retards the rate of deacetylation of pluripotency genes activated by DNA damage

**Figure 5.**
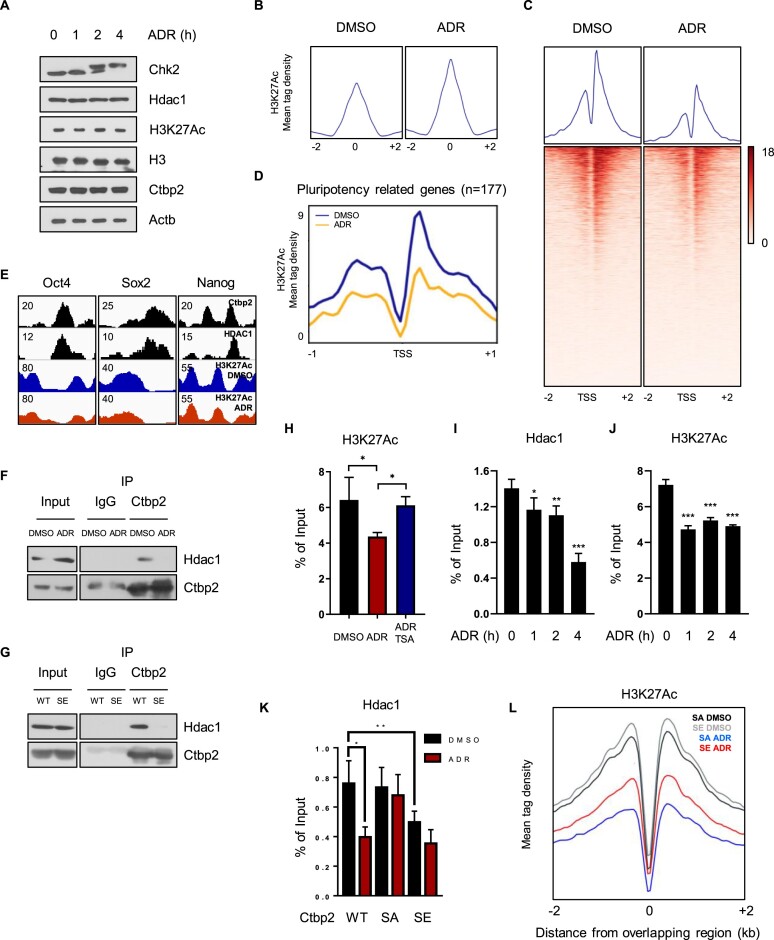
Phosphorylated Ctbp2 retards the rate of deacetylation of pluripotency genes. (**A**) Western blot images with 500 nM ADR treatment over the indicated time. (**B**–**D**) Analysis of the mean tag density of H3K27 acetylation: (B), at the overall H3K27 acetylation peaks; (C), at the transcriptional start sites of Ctbp2 target genes; and (D), at the transcriptional start sites of pluripotency genes, with or without 500 nM ADR treatment (GO: 0019827). (**E**) An IGV snapshot showing overlaps between peak files of Ctbp2, Hdac1 and H3K27Ac. (**F**) The immunoprecipitation (IP) assay was performed using anti-Ctbp2 antibodies with or without 500 nM ADR. (**G**) Immunoprecipitation results for Hdac1 with wild-type or S164E KI mESCs. IP was conducted with anti-Ctbp2 antibodies. (**H**) H3K27Ac ChIP at the Oct4 enhancer following 4 h of 500 nM ADR treatment with or without 1 μM TSA (*n* = 4, *P < 0.05). (**I**) Chromatin immunoprecipitation of Hdac1 on the Oct4 enhancer with 500 nM ADR for the indicated time (*n* = 4, *P < 0.05, **P < 0.01 and ***P < 0.001). (**J**) ChIP analysis of H3K27Ac on the Oct4 enhancer with 500 nM ADR for the indicated time (*n* = 4 and ***P < 0.001). (**K**) Hdac1 ChIP with wild-type or S164A or S164E mESCs with or without 500 nM ADR (*n* = 5, *P < 0.05, **P < 0.01). (**L**) H3K27 acetylation ChIP profiling for Ctbp2 and Hdac1 overlapping regions in S164A or S164E mESCs with or without 500 nM ADR.

HDACs are recruited at the site of damage, followed by deacetylation, to repress transcription (17), and Hdac1, along with the NuRD complex, co-occupies Ctbp2 target genes such as Oct4, Sox2 and Nanog to catalyze H3K27 deacetylation during differentiation (45,46) (Supplementary Figure S5). Because we observed Ctbp2 release, we verified the role of phosphorylation on damage-mediated deacetylation if H3K27Ac histone marks could be altered in this situation. Although total H3K27 acetylation levels were slightly increased under this condition (Figure 5A and B), H3K27 acetylation levels decreased near the transcription start site of Ctbp2 and pluripotency genes (Figure 5C and D). Oct4, Sox2 and Nanog presenting reduced H3K27Ac levels (Figure 5E). Since Ctbp2 colocalized with the NuRD complex to standby silencing pluripotency genes (Supplementary Figure S5), we compared the binding affinity of S164E Ctbp2 to Hdac1. The IP assay confirmed that Hdac1 was segregated from Ctbp2 based on phosphorylation (Figure 5F and G). Trichostatin A (TSA), an HDAC inhibitor, effectively offset H3K27 deacetylation (Figure 5H), and the rate of H3K27Ac reduction slowed down after Hdac1 release, indicating that this phenomenon occurred through Hdac1 activity (Figure 5I and J). During this process, Hdac1 separated from chromatin in proportion to the phosphorylation levels of Ctbp2. Nonphosphorylated S164A Ctbp2, which consistently bound to chromatin, kept Hdac1 bound to chromatin tightly upon damage. By contrast, S164E Ctbp2 ESCs showed low binding affinity of Hdac1 (Figure 5K). Therefore, in S164E Ctbp2 ESCs, H3K27Ac levels were maintained during damage (Figure 5L). Consequently, in ESCs, pluripotency genes were immediately deacetylated upon damage, but phosphorylation-mediated Ctbp2 detachment decreased this effect.

### Phosphorylated Ctbp2 binds PRC2 through Suz12

**Figure 6.**
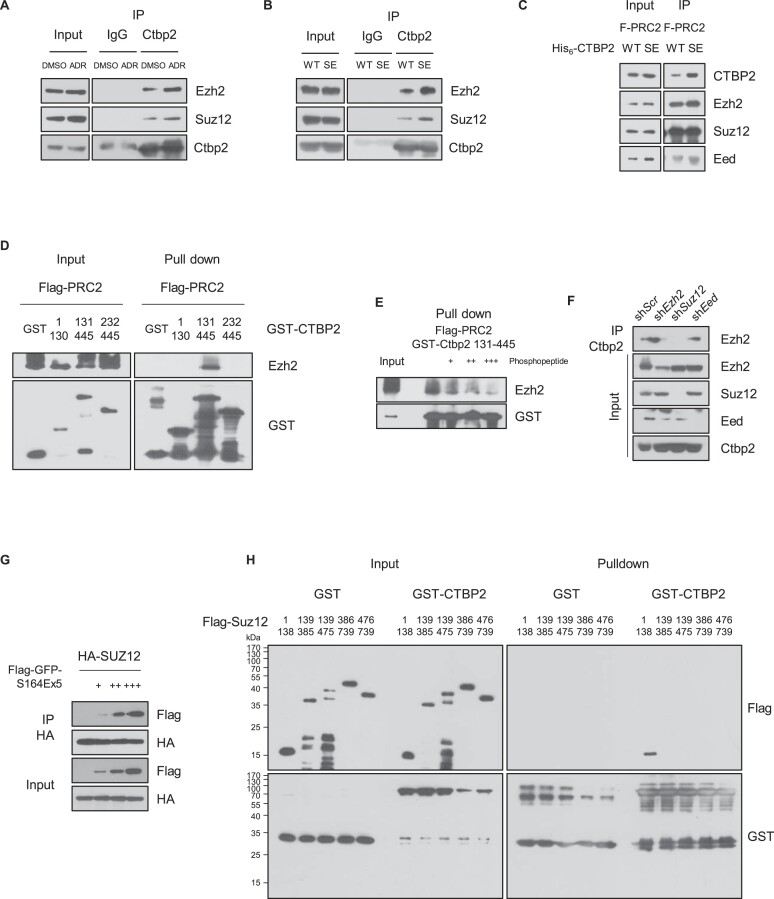
Phosphorylated Ctbp2 interacts with PRC2 through Suz12. (**A**) Immunoprecipitation assay. IP was performed with anti-Ctbp2 antibodies with or without 500 nM ADR. (**B**) IP was performed with anti-Ctbp2 antibodies in wild-type or S164E KI mESCs. IgG was used for negative control. (**C**) *In vitro* binding assay using 1 μg of FLAG-PRC2 and His_6_-Ctbp2 each, precipitated with FLAG M2 beads. (**D**) Domain mapping to identify binding sites on Ctbp2 for PRC2 interaction, using truncated forms of Ctbp2 incubated with the FLAG-PRC2 complex. The pull-down was conducted using glutathione Sepharose. (**E**) Competition assay. Ctbp2 160–173 amino acids (serine 164 phosphorylated) peptides were added in a dose-dependent manner with GST-CTBP2 and FLAG-PRC2 pull downs. (**F**) Immunoprecipitation assay in mESCs with lentiviral-mediated knockdown of Ezh2, Suz12 or Eed using anti-Ctbp2 antibodies. shScr was used as a negative control. (**G**) Suz12 and FLAG-GFP-Ctbp2 S164E × 5 peptide (160–173 amino acids) were transfected into HEK293T cells. Anti-HA antibodies were used for IP. (**H**) Recombinant truncated FLAG-Suz12 and GST-CTBP2 were incubated for the *in vitro* pull-down assay. Pull down was performed with glutathione Sepharose. GST was used as a negative control.

To orchestrate epigenetic modulation during silencing, Ctbp2 works with NuRD, followed by PRC2 (26). Under DNA damage conditions, epigenetic silencing occurred through histone methylation (47). Because we confirmed that phosphorylated Ctbp2 separated from the NuRD complex and retarded deacetylation of pluripotency genes, we tested its binding ability to PRC2. In contrast to that with the NuRD complex or ZFPs, phosphor-Ctbp2 preferred interacting with PRC2 in the IP assay (Figure 6A and B). The *in vitro* binding assay confirmed that wild-type Ctbp2 directly binds PRC2 (Ezh2/Suz12/Eed/RbAp46), and the phosphomimetic modification increased their interactions (Figure 6C). To determine the domain that binds PRC2, we truncated Ctbp2 into three parts (1–130, 131–445 and 232–445 amino acids). Only the domain containing amino acids 131–231 interacted with PRC2 (Figure 6D). Indeed, a phosphorylated Ctbp2 peptide (160–173 amino acids containing phosphorylated serine 164), which is located on the Ctbp2 tetramer surface, competed for their interactions in a dose-dependent manner (Figure 6E). This indicates that serine 164 phosphorylation is important for their binding. Next, we conducted lentiviral knockdown of each PRC2 core gene to identify the component crucial for their interactions. When *Suz12* was knocked down, Ctbp2 failed to bind to the PRC2 complex (Figure 6F). We designed a 5 × repeat Ctbp2 S164E (160–173 amino acids containing the S164E mutation) construct following FLAG-GFP (FLAG-GFP-S164E × 5) to elucidate their interaction. The IP assay showed that Suz12 binds to the Ctbp2 160–173 amino acid repeat peptide (Figure 6G). Therefore, we split Suz12 into five parts based on the functional domains (1–138, 139–385, 139–475, 386–739 and 476–739 amino acids). The *in vitro* binding assay showed that Ctbp2 directly binds to the N-terminal region (1–138 amino acids) of Suz12 (Figure 6H).

### Phosphorylated Ctbp2 competes with Jarid2 and inhibits H3K27me3 formation

**Figure 7.**
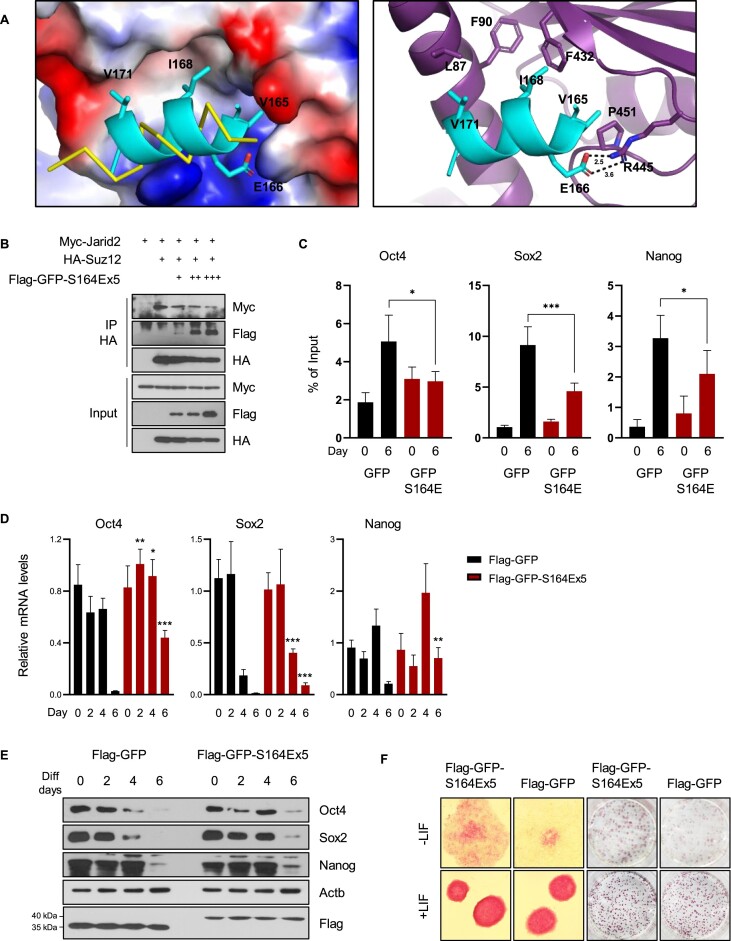
Phosphorylated Ctbp2 competes with Jarid2 and inhibits H3K27me3 formation. (**A**) Interactions between PRC2 and the Ctbp2 peptide in the *in silico* complex model. The structure of PRC2 is shown as a surface representation with electrostatic potentials (left) and as a cartoon (right).For clarity, only the helices of Ctbp2 (residues 164–173) and Jarid2 (residues 155–165; PDB code: 6NQ3) (51) are presented as a cyan cartoon and a yellow ribbon (left), respectively. The interacting residues between PRC2 and the Ctbp2 peptide are depicted as sticks with labels. Salt bridges are represented as dashed lines with labeled distances (Å). Nitrogen and oxygen atoms in all sticks are colored in blue and red, respectively. (**B**) Competition assay. HA-Suz12 and Myc-Jarid2 were transfected into HEK293T cells. FLAG-GFP-Ctbp2 S164E (160–173 amino acids) ×5 was transfected in a dose-dependent manner. (**C**) ChIP for H3K27me3 was conducted in mESCs overexpressing FLAG-GFP or FLAG-GFP-Ctbp2 S164E (160–173 amino acids) ×5, targeting the Oct4, Sox2 enhancer and Nanog promoter (*n* = 5, *P < 0.05 and ***P < 0.001). (**D** and **E**) Pluripotency gene expression was assessed during differentiation, which was induced by withdrawing LIF for specific time periods. Messenger RNA (mRNA) (*n* = 4, *P < 0.05, **P < 0.01 and ***P < 0.001) and protein levels were compared between Flag-GFP control and Flag-GFP-S164Ex5 cell lines. (**F**) Alkaline phosphatase staining; 10× magnified or original images of alkaline phosphatase-positive colonies. Differentiation was induced for 6 d.

The N- terminus of Suz12 is a binding site for Jarid2 (48). This interaction is crucial for PRC2 function in ESCs (49,50). We hypothesize that phosphorylated Ctbp2 competes with Jarid2, potentially altering PRC2 function. To elucidate the competition between Ctbp2 and Jarid2 for binding to the Suz12 subunit of PRC2, we performed *in silico* complex modeling for the PRC2/Ctbp2 peptide (residues 160–173) using AlphaFold 3(42). As shown in Figure 7A (left), the Ctbp2 helix (residues 165–173) binds to the same site on Suz12 as the Jarid2 peptide (residues 147–165), as observed in the crystal structure of the PRC2/Jarid2 peptide complex (PDB code: 6NQ3) (51). The Ctbp2 helix, which includes hydrophobic residues Val165, Ile168 and Val171, fits into the hydrophobic pocket clustered by Leu87, Phe90, Phe432 and Pro451 in Suz12. Additionally, Glu166 of Ctbp2 helps position the helix through a salt bridge interaction with Arg445 of Suz12 (Figure 7A, right). In the case of tetrameric Ctbp2, the key residues Val165, Glu166, Ile168 and Val171 exhibit %BSA values (the ratio of buried surface area to solvent-accessible surface area, calculated by PDBe PISA v1.52) (39,52,53) of 98.1, 58.7, 97.3 and 18.7%, respectively, indicating their primary involvement in the tetramerization of Ctbp2. Therefore, in the tetrameric state, these four residues cannot mediate binding to PRC2. Remarkably, Val165, Glu166, Ile168 and Val171 exhibit %BSA values of 0% in the case of monomeric Ctbp2, indicating that they are fully exposed to solvent and thus ready to bind to PRC2. Consequently, the phosphorylation of Ser164, which mediates the transition of tetrameric Ctbp2 to monomeric Ctbp2, is responsible for its association with PRC2. To test whether Ctbp2 interrupts the Jarid2–PRC2 interaction, Ctbp2 peptides were cotransfected with these proteins. The interactions between Suz12 and Ctbp2 increased in a dose-dependent manner, whereas the binding of Jarid2 decreased (Figure 7B). Disruption of Jarid2 impaired the ability of PRC2 to catalyze H3K27me3 on pluripotency genes, even in the presence of differentiation signals (Figure 7C). As a result of the reduced H3K27me3 levels, cells expressing the Ctbp2 peptide showed higher transcription of core pluripotency genes such as Oct4, Sox2, and Nanog during differentiation (Figure 7D), and maintained elevated protein levels compared to the control (Figure 7E). Consequently, cellular differentiation was retarded (Figure 7F). These results suggest that phosphorylated Ctbp2 disrupts the function of PRC2 by competing with Jarid2, leading to altered epigenetic regulation and impaired differentiation of stem cells.

## Discussion

Cells receive various forms of damage, including replication stress, reactive oxygen species, ultraviolet stress, alkylating reagents and chemicals, which can cause deleterious mutations. During stress, cells arrest the cell cycle and repair the damage (2). In case of excessive damage, cells turn on the apoptosis pathway to avoid propagation of undesirable DNA lesions or start differentiation (4,54).

Because ESCs continuously proliferate and can differentiate into most cell types, the threats should be controlled. According to previous studies, ESCs have a strong damage repair system. For example, high fidelity homologous recombination is preferred over the error prone nonhomologous end joining when double strand DNA breaks occur in the ESCs (55). In addition, the ESCs retain high DNA repair capacity (56). However, at the same time, it is believed that pluripotency genes, such as Oct4, Sox2 and Nanog, are getting decreased even before cells tilt their fate to apoptosis. Ironically, they would be in trouble to maintain pluripotency when damage is recovered because pluripotency genes might be already lacking. In this regard, ESCs should have an authoritative mechanism to determine if they can preserve their original character.

Here, we demonstrated that activated CHK2 phosphorylates Ctbp2, leading to the disruption of Ctbp2 tetramers and altering the composition of the transcriptional corepressor complex. Consistent with our findings, other studies focusing on monomeric Ctbp2 have shown that the epigenetic modifiers associating with Ctbp2 are determined by the tetrameric state of Ctbp2 (43,57,58,59,60). This influences the functional dynamics of the Ctbp2 complex, highlighting the significance of the structural states of Ctbp2 in regulating its interactions and the subsequent transcriptional repression activities.

Investigation of the process revealed that prior to changes in Ctbp2 binding partners, HDAC1 swiftly deacetylates in response to DNA damage. However, what activates HDAC1 under these conditions is unclear. Several studies have shed some light. ADR was found to increase the transcriptional profile of HDAC expression (61). Ionizing radiations strengthened the interaction between ATM and HDAC1, increasing HDAC1 activity (62). Because the ATM phosphorylation motif (pS/T–Q–G) is well conserved at the N-terminus of HDAC1, HDAC1 activation might correlate reciprocally with damage states.

We previously reported that Ctbp1 binds to p300 acetyltransferase through the bromodomain and represses its activity. This interaction has a reciprocal correlation with the tetrameric state of Ctbp1 (59). Therefore, monomeric Ctbp1 blocked the transactivation function of p300. Because Ctbp2 target pluripotency genes are deacetylated by HDAC1, the subsequent phosphorylation of Ctbp2 could help sequester p300 until DNA damage is managed.

LSD1, an H3K4me1/2 demethylase, is an important component of the NuRD complex (63). Similar to HDAC1, LSD1 separates from phosphorylated Ctbp2. However, we found that H3K4me1 levels on Ctbp2 target regions were not altered (Supplementary Figure S6). Although many studies have shown that LSD1 is indispensable to stemness (12) and is needed during the DNA damage response (64), we found no evidence that LSD1 controls pluripotency against damage in this context. H3K4me1 and H3K27Ac coexist in enhancer regions. However, although even it is still controversial, the importance of H3K27Ac to enhancer activity in mESCs is less essential (65). Given that the temporal reduction of H3K27Ac would not immediately inhibit enhancer activity, it could imply that Ctbp2 only give a chance to hold the situation but not promptly convert ESC identity by decreasing H3K27Ac levels but not H3K4me1 levels and, wait and see how menacing this situation is. Subsequent loss of ability of monomeric Ctbp2 to repress DDR genes underpins this phenomenon (66).

PRC2 oversees transcriptional silencing by catalyzing histone methylation. Ctbp2 was reported to cooperate with PRC2 during differentiation through unclear mechanisms. Notably, the core components of PRC2 (Ezh2, Suz12 and Eed) do not have typical Ctbp2-binding motifs (PXDLS or RRT). A58E edited Ctbp2, deficient in PXDLS-binding ability, interacted with PRC2, indicating that a typical domain is not required for this interaction (Supplementary Figure S7A). Consistently, the PXDLS-binding motif (N-terminus of Ctbp2: 33–127 amino acids) was not required for Suz12 binding (Figure 6D), indicating other binding mechanisms. Both wild-type and phosphorylated Ctbp2 interact with PRC2, but phosphorylated Ctbp2 exhibits enhanced binding capacity. G189A Ctbp2 is a monomeric mutant that has low NADH-binding affinity. G189A Ctbp2 shows the same binding effect to PRC2 as wild-type Ctbp2, indicating that phosphorylation on serine 164 not only contributes to monomeric transition but also increases binding affinity (Supplementary Figure S7B).

PRC2 can form various versions of the repressor complex depending on the bound accessory protein (67). Jaird2 is one of the accessory subunits crucial for forming PRC2.2. In ESCs, Jarid2 shares target genes with PRC2 (50,68), and disruptions, such as the loss of Jarid2 or mutations in the N-terminal of Suz12, lead to imprecise PRC2 recruitment (69,70). In lineage committed cells, the N-terminal of Jarid2 is translationally truncated (71). Considering Ctbp2 shares the Suz12 binding site with Jarid2, they might confer distinct roles to PRC2 by changing binding partners in response to the cellular contexts. Although we found that the 160–173 amino acid region of Ctbp2 is crucial for binding to Suz12, another accessory component called PALI1/2 can interact with Ctbp (72). In addition, RbAp46/48 has a typical Ctbp binding motif (PXDLS). Therefore, Ctbp2 may interact with PRC2 in multiple ways.

Ctbp is conserved in many species. The vertebrate genome encodes Ctbp1 and Ctbp2. Both have high sequence similarity (78% in amino acids) and display functional redundancy as well as unique properties depending on the pathways they are involved in (73). Serine 164 of Ctbp2 corresponds with serine 158 of Ctbp1. Phosphorylation of serine 158 of Ctbp1 by AMPK or PAK altered the sublocation of Ctbp1 and depressed its corepressor function in HEK293T and MCF-7 cells, respectively (74,75). Consistently, we confirmed that AMPK phosphorylates serine 164 of Ctbp2 *in vitro* (Supplementary Figure S3D). However, AMPK failed to phosphorylate Ctbp2 in cells (Supplementary Figure S3E). Considering the complexity of the cellular system, *in vivo* systems are intricate. For example, metformin, an AMPK activator, stabilizes the Ctbp2 tetramer by increasing the NADH/NAD^+^ ratio rather than destabilizing it (66). Nutrition status and optimal metabolic state are as indispensable for pluripotency. Therefore, there would be other mechanisms to protect ESCs under each type of stress. In conclusion, ESCs possess epigenetically protective mechanisms to bethink itself of the identity against DNA damage.

It is well known that Ctbp2 loss leads to embryonic lethality at E10.5. Ctbp2-null embryos exhibit delayed neural development and heart formation abnormalities, which are believed to be linked to defects in both placenta and yolk sac formation. These placental defects in Ctbp2-null embryos arise after chorioallantoic fusion and the initiation of branching (28). The novel functions of Ctbp2 in protecting stem cell properties might indeed be connected to the embryonic lethality observed in global Ctbp2 knockout models. Our previous work demonstrated that knockdown of Ctbp2 or knockout of the ZFPs, ZFP217 and ZFP516, which leads to Ctbp2 mislocalization, resulted in reduced H3K27me3 levels on pluripotency genes during differentiation (26,44). We demonstrated the relevance of Ctbp2 and PRC2 in this study. It is well established that abnormal H3K27me3 regulation leads to embryonic lethality (76). The loss of PRC2 components, such as EZH2 (E6.5 to E8.5), Suz12 (E7.5 to E8.5) and EED (E7.5 to E8.5), results in deficient gastrulation and also causes embryonic lethality at early stages (77–79). Jarid2 deficiency leads to neural tube and cardiac defects, resulting in embryonic lethality around E15.5 (80–82). These studies suggest that Ctbp2 has a distinct role in regulating H3K27me3, and that alterations in H3K27me3 due to Ctbp2 dysfunction or modifications could contribute to the embryonic lethality phenotype, given the critical role of Ctbp2, PRC2 and H3K27me3 in development.

## Supplementary Material

gkae1076_Supplemental_File

## Data Availability

High-throughput sequencing data for ChIP-sequencings generated during this study are available at Gene Expression Omnibus (GEO number: GSE271772). Raw HDAC1 ChIP-sequencing data were obtained from a publicly available data GSM687277 and reanalyzed in this study. Raw MTA1 and MTA2 ChIP-sequencing data were obtained from a publicly available data GSE122833 and reanalyzed in this study. Raw MBD2 and MBD3 ChIP-sequencing data were obtained from a publicly available data GSE39610 and reanalyzed in this study. Protein three dimensional structures were obtained from Protein Data Bank. PDB number of Ctbp2 structure: 6WKW and 2OME. PDB number of Jarid2 and Suz12 structure: 6NQ3.
