# Supplementary material for: Phosphorylation-mediated disassembly of C-terminal binding protein 2 tetramer impedes epigenetic silencing of pluripotency in mouse embryonic stem cells
Source: Nucleic Acids Res. 2024 Nov 26;52(22):13706–22. doi: 10.1093/nar/gkae1076 (PMC11662665; doi:10.1093/nar/gkae1076)
Supplement: gkae1076_Supplemental_File [file gkae1076_supplemental_file.pdf]

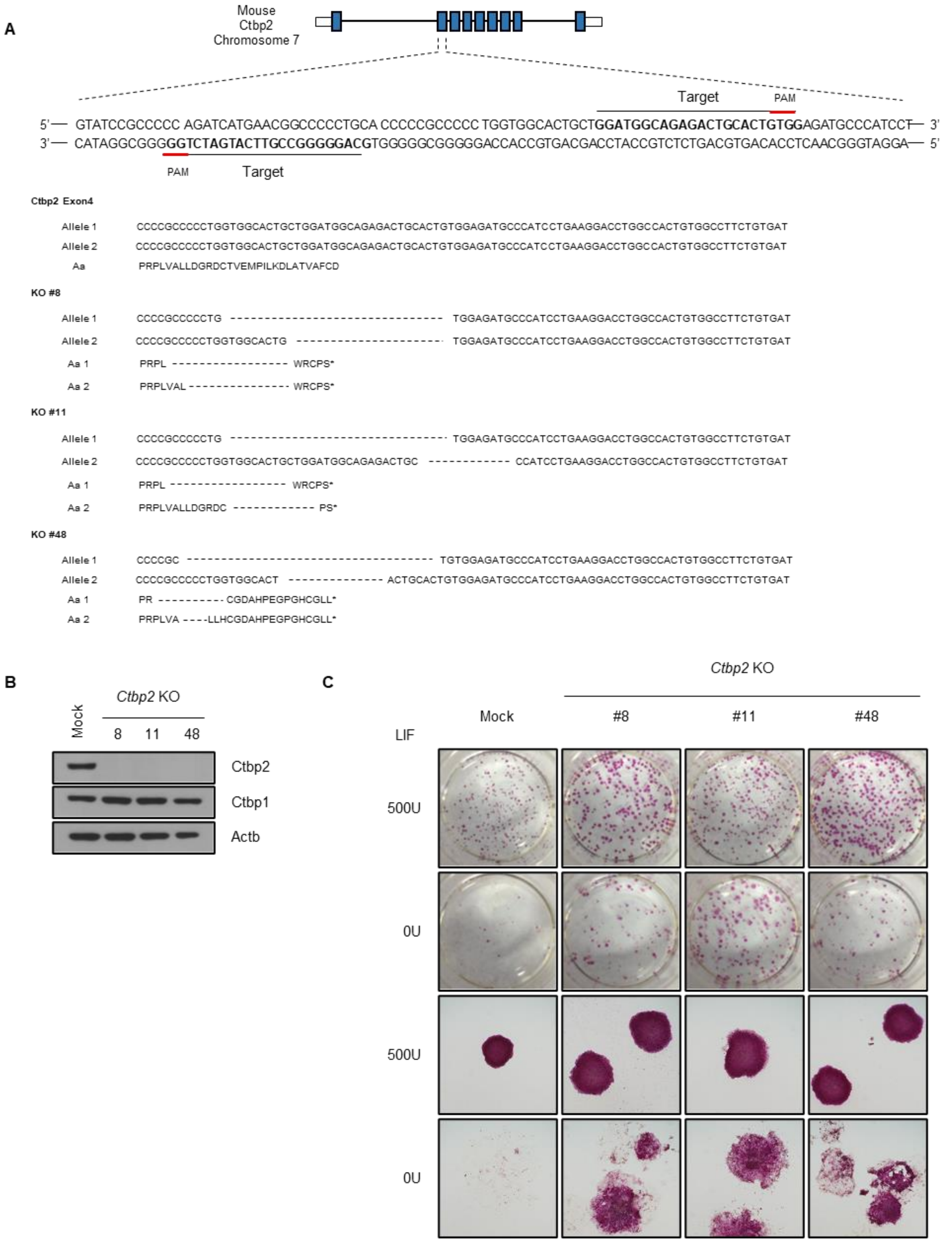

Supplementary Figure S1. The establishment of Ctbp2 knockout cell lines (A) Schematic representation of the CRISPR/Cas9 system used for Ctbp2 knockout. (B) Western blot images of Ctbp2 knockout. (C) Alkaline phosphatase staining comparing wild-type and Ctbp2 knockout cell lines.

A

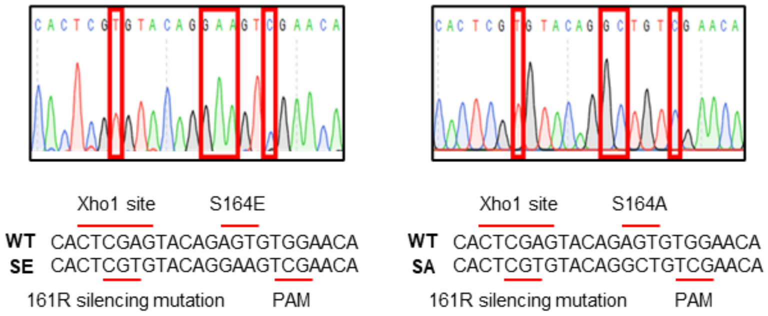

Supplementary Figure S2. Sequencing results of S164E or S164A Ctbp2 genomic knock-in (A) Sanger sequencing results showing the substitution of serine 164 with either glutamic acid or alanine.

A

Symbol	Accession number	Entrez Gene Name
PRKAA1	IPI00556823	protein kinase, AMP-activated, alpha 1 catalytic subunit
RPS6KA1	IPI00114332	ribosomal protein S6 kinase, 90kDa, polypeptide 1
CHK1	IPI00387390	checkpoint kinase 1
CHK2	IPI00130553	checkpoint kinase 2
BUB1	IPI00882019	BUB1 mitotic checkpoint serine/threonine kinase
CAMK2B	IPI00470962	calcium/calmodulin-dependent protein kinase II, beta
CAMK2G	IPI00228045	calcium/calmodulin-dependent protein kinase II gamma
CAMK2D	IPI00112584	calcium/calmodulin-dependent protein kinase II delta
STK38	IPI00126943	serine/threonine kinase 38
EIF2AK3	IPI01007978	eukaryotic translation initiation factor-2 alpha kinase 3
SRPK1	IPI00387234	SRSF protein kinase 1

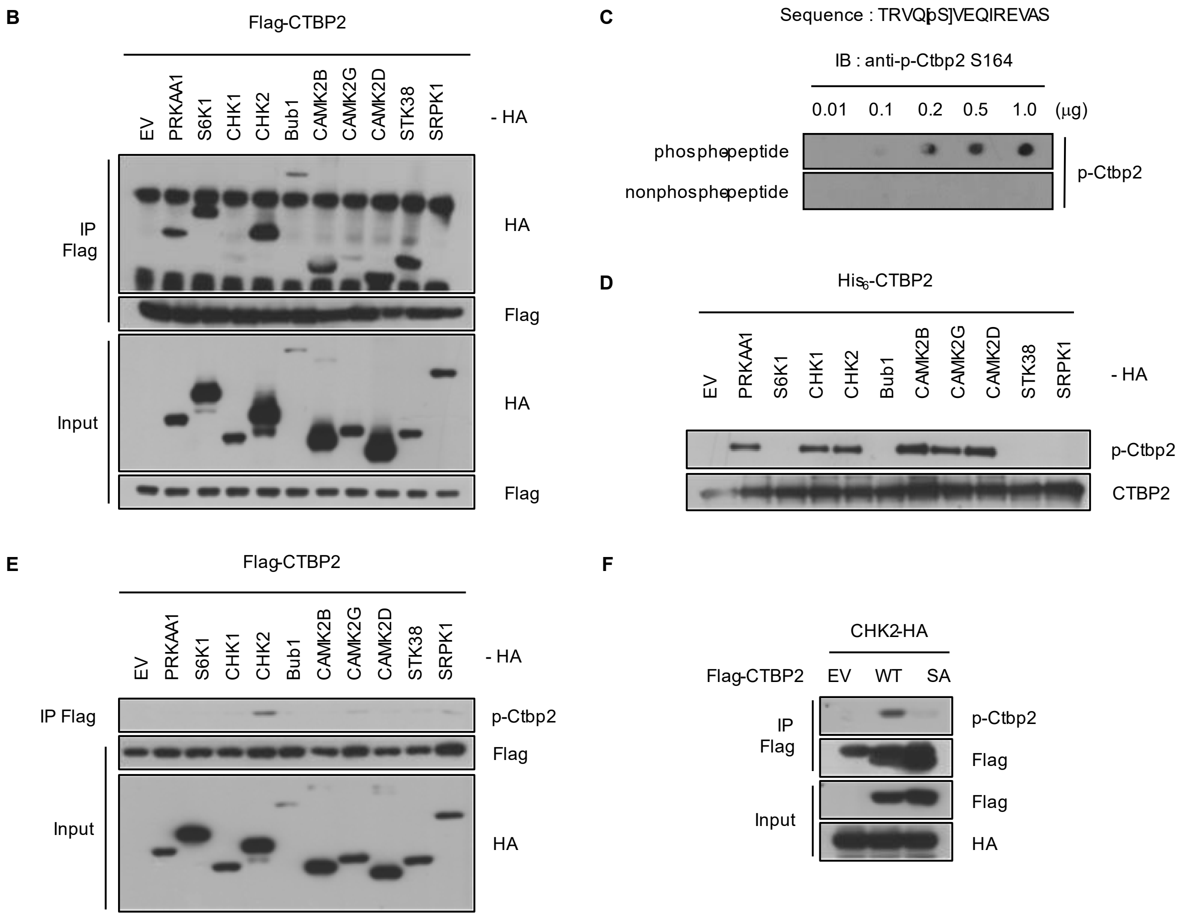

Supplementary Figure S3. AMPK, CHK and CAMK2 phosphorylate serine 164 of Ctbp2 *in vitro* (A) Potential kinase list for phosphorylating serine 164 of Ctbp2. (B) Immunoprecipitation assay. FLAG-Ctbp2 and kinase candidates were cotransfected into HEK293T cells and immunoprecipitated with anti-FLAG antibodies. (C) Phospho-Ctbp2 S164 antibody test. Dot blot conducted with the indicated amounts of phosphorylated and non-phosphorylated peptides. (D) *In vitro* phosphorylation assay. HA-kinases were transfected into the HEK293T cells. Kinases were subsequently immunoprecipitated

using anti-HA antibodies in conjunction with Protein G Plus-Agarose beads. Bead bound kinases were incubated with His_6-Ctbp2 recombinant proteins to perform the phosphorylation assay. (E) FLAG-Ctbp2 and kinase candidates were cotransfected into HEK293T cells. Immunoprecipitation was performed with anti-FLAG antibodies. (F) Chk2-HA and FLAG-Ctbp2 wild-type or S164A cotransfected into HEK293T cells and immunoprecipitated with anti-FLAG antibodies.

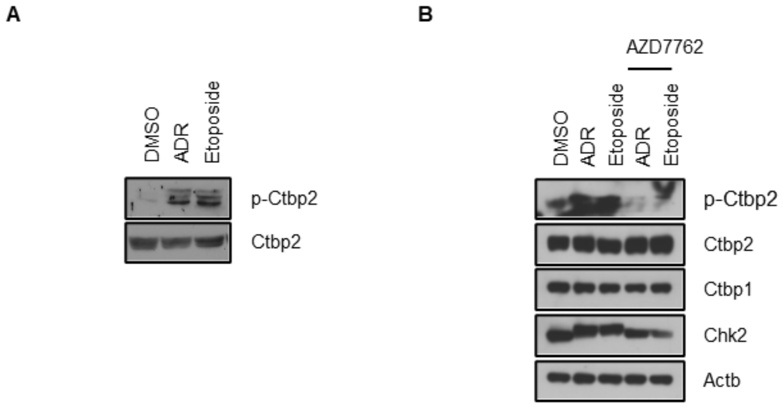

Supplementary Figure S4. Adriamycin and etoposide induce the phosphorylation of Ctbp2 (A) 500 nM adriamycin or 10 μM etoposide was treated in E14 ESCs. (B) E14 ESCs were pretreated with 10 μM of the CHK2 inhibitor AZD7762 for 2 hours before treatment with adriamycin and etoposide.

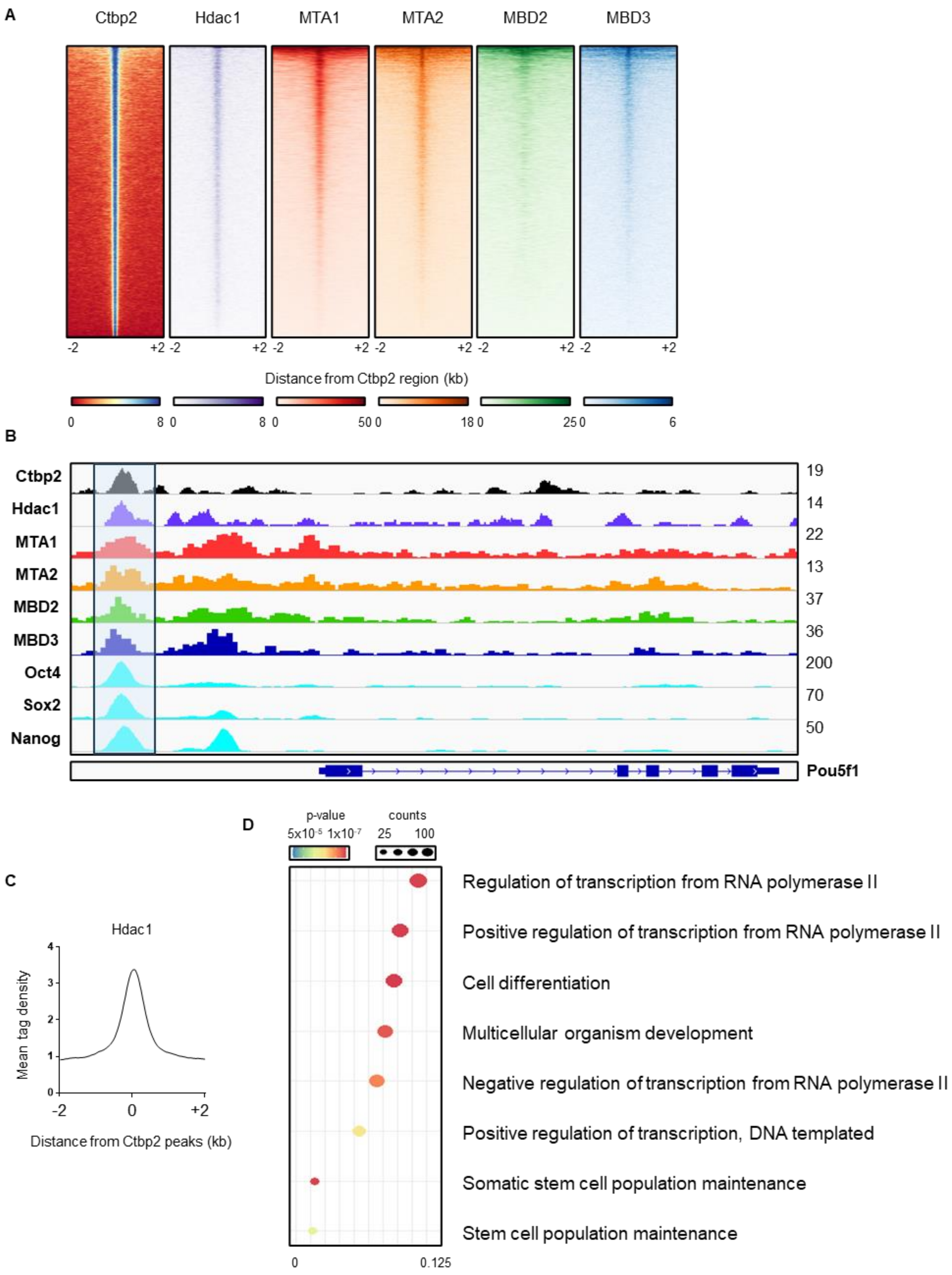

Supplementary Figure S5. NuRD complex is co-occupied with Ctbp2 on pluripotent genes (A) Heat map plots showing the distribution of Ctbp2, HDAC1, MTA1, MTA2, MBD2, and MBD3 at Ctbp2 binding

regions. (B) Integrative Genomics Viewer (IGV) snapshot illustrating the co-localization of Ctbp2 and the NuRD complex at the pou5f1 enhancer. (C) Mean tag density of HDAC1 at Ctbp2 peak regions. (D) Gene ontology analysis of genes targeted by both Ctbp2 and HDAC1.

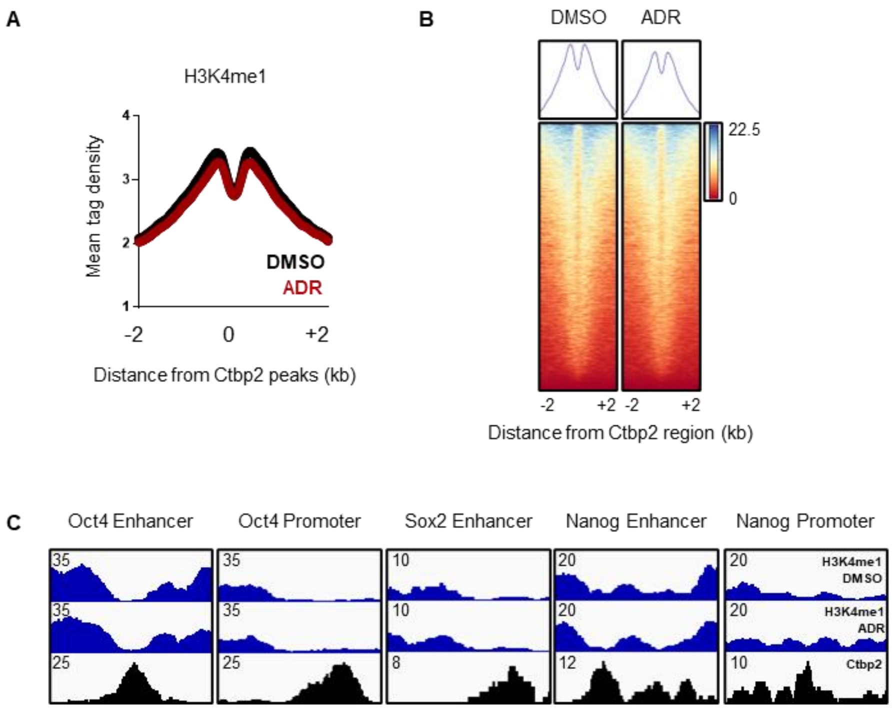

Supplementary Figure S6. DNA damage does not change H3K4me1 levels on Ctbp2 target genes (A) Mean tag density of H3K4me1 at Ctbp2 peaks with or without 500 nM adriamycin treatment. (B) Heat map plot of H3K4me1 at the Ctbp2 regions. (C) IGV snapshot of H3K4me1 at Ctbp2 target genes.

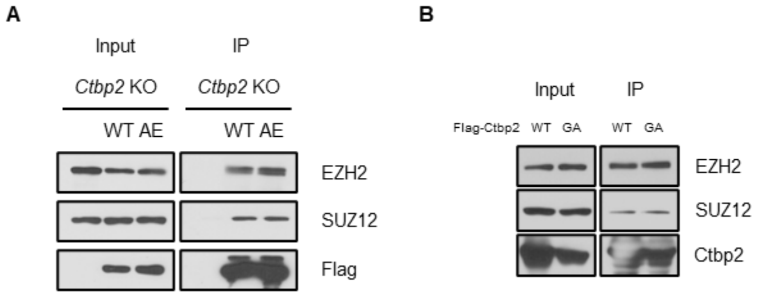

Supplementary Figure S7. Monomeric Ctbp2 and PXDLS-binding deficient Ctbp2 also bind to PRC2 (A) Immunoprecipitation was conducted in FLAG-wild-type or FLAG-AE-Ctbp2 overexpressing Ctbp2 knockout E14 cells using anti-FLAG antibodies. (B) FLAG- wild-type or FLAG-GA-Ctbp2 was transfected into HEK293T cells. Immunoprecipitation was conducted using anti-FLAG antibodies.

**Primer lists for site directed mutagenesis**	Sense (5' to 3')	Anti-sense (5' to 3')
S106E	atcgtgcggataggcGAAggctatgacaacgt	acgttgtcatagccTTCgcctatccgcacgat
S164E	cacgcgggttcagGAGgtggagcagatccgcg	cgcggatctgctccacCTCctgaacccgcgtg
S424E	acagtggcacatcctGAAcaagcgccctctcc	ggagagggcgcttgTTCaggatgtgccactgt
S428E	ccttcccaagcgcccGAAcccaaccagcccac	gtgggctggttgggTTCgggcgcttgggaagg
**single guide RNA for Ctbp2**		
KO#1	CACCG GGATGGCAGAGACTGCACTG	AAAC CAGTGCAGTCTCTGCCATCC C
KO#2	CACCG GCAGGGGGCCGTTCATGATC	AAAC GATCATGAACGGCCCCCTGC C
S164E and S164A	CACCG AGGCACTCGAGTACAGAGTG	AAAC CACTCTGTACTCGAGTGCCT C
**HDR template**		
S164E HDR template	GAG GCC CAG TGT TTC CCC TCG GAT CCG AGC AGC TCC TGA GGC GAC CTC ACG GAT CTG TTC GAC TTC CTG TAC ACG AGT GCC TTC TCG GAG GGC CTG GTA GAG CCA TGT GTT CCG CCG ATA CAG ATT GAG A	
S164A HDR template	GAG GCC CAG TGT TTC CCC TCG GAT CCG AGC AGC TCC TGA GGC GAC CTC ACG GAT CTG TTC GAC AGC CTG TAC ACG AGT GCC TTC TCG GAG GGC CTG GTA GAG CCA TGT GTT CCG CCG ATA CAG ATT GAG A	
**Primer lists for ChIP**	Forward (5' to 3')	Reverse (5' to 3')
mouse Oct4 enhancer	CTTGAACTGTGGTGGAGAGTGCTG	TAAGGAAGGGCTAGGACGAGAGG
mouse Sox2 enhancer	TTAGGGTAAGGTACTGGGAAG	TTCCTATGTGTGAGCAAGAAC
mouse Nanog promoter	ATGAGTGTGGGTCTTCCTG	TGATGAGGCGTTCCCAGAA

Supplementary table S1. Oligomer list for this study
